# Cell density-dependent proteolysis by HtrA1 induces translocation of zyxin to the nucleus and increased cell survival

**DOI:** 10.1038/s41419-020-02883-2

**Published:** 2020-08-21

**Authors:** Fabio Sabino, Elizabeta Madzharova, Ulrich auf dem Keller

**Affiliations:** 1 Technical University of Denmark, Department of Biotechnology and Biomedicine, Søltofts Plads, 2800 Kongens Lyngby, Denmark; 2 ETH Zurich, Department of Biology, Institute of Molecular Health Sciences, Otto-Stern-Weg 7, 8093 Zurich, Switzerland

**Keywords:** Biochemistry, Proteolysis

## Abstract

Proteases modulate critical processes in cutaneous tissue repair to orchestrate inflammation, cell proliferation and tissue remodeling. However, the functional consequences and implications in healing impairments of most cleavage events are not understood. Using iTRAQ-based Terminal Amine Isotopic Labeling of Substrates (TAILS) we had characterized proteolytic signatures in a porcine wound healing model and identified two neo-N termini derived from proteolytic cleavage of the focal adhesion protein and mechanotransducer zyxin. Here, we assign these proteolytic events to the activity of either caspase-1 or serine protease HtrA1 and analyze the biological relevance of the resultant zyxin truncations. By cellular expression of full-length and truncated zyxin proteins, we demonstrate nuclear translocation of a C-terminal zyxin fragment that could also be generated in vitro by HtrA1 cleavage and provide evidence for its anti-apoptotic activities, potentially by regulating the expression of modulators of cell proliferation, protein synthesis and genome stability. Targeted degradomics correlated endogenous generation of the same zyxin fragment with increased cell density in human primary dermal fibroblasts. Hence, this newly identified HtrA1-zyxin protease signaling axis might present a novel mechanism to transiently enhance cell survival in environments of increased cell density like in wound granulation tissue.

## Introduction

Cutaneous wound healing is a complex tissue response that is initiated upon skin injury to restore the skin’s barrier function, which is critical to protect the human body from environmental insults^1^. Proteases play major roles in all phases of wound healing by contributing to formation of the blood clot, initiating innate immune responses through complement activation, facilitating migration of immune cells, keratinocytes and fibroblasts and remodeling of the extracellular matrix during scar formation. Importantly, proteases have also been identified as major contributors to chronic non-healing wounds, which do not progress through the normal healing process, but are stalled in a persistent inflammatory state, with impaired neovascularization and reduced synthesis of collagen^2–4^. Thus, identification and mechanistic dissection of proteolytic events in wound repair are critical to better understand this important tissue response and to devise novel strategies for intervention with aberrant protease activities in impaired healing.

In recent studies, we characterized proteolytic signatures during wound healing by iTRAQ-based Terminal Amine Isotopic Labeling of Substrates (TAILS) of wound exudates from a clinically relevant porcine wound healing model and from trauma patients^5,6^. By N-terminomics analysis of liquid biopsies collected from the porcine model, we revealed 1667 neo-N termini from 492 proteins. Among these we identified two neo-N termini in the focal adhesion (FA) protein zyxin indicating proteolytic processing.

Zyxin has three LIM domains localized in the C-terminal region that tether the protein to FAs^7^. LIM domains are cysteine-rich sequences that adopt zinc finger conformations, which allow protein-protein or protein-DNA interactions. LIM domains containing proteins have been associated with important biological functions, such as response to force in the actin cytoskeleton^7–9^. More than 20 LIM domain proteins have been identified in FAs, whereby zyxin has received special attention due to its roles in actin dynamics and nuclear-cytoplasm communication^7,10^. The central region of the zyxin protein harbors two leucine-rich nuclear export sequences (NES)^7^. Mutation of these NES sequences or the treatment of cells with leptomycin B (an inhibitor of Crm1-dependent nuclear export) lead to accumulation of zyxin in the nucleus, showing a role of zyxin in signal transduction between focal adhesions and the nuclear compartment^11^. However, the mechanism of zyxin translocation to the nucleus is poorly characterized. As zyxin does not have a canonical nuclear localization signal (NLS), shuttling between focal adhesions and the nucleus should be mediated by unconventional mechanisms or through association with NLS containing proteins^12^. Although the role of zyxin in the nucleus is still unclear, evidences obtained from other members of the zyxin family migrating to the nucleus support the idea that these proteins interact with transcription factors or the basal transcription machinery to regulate gene expression^12–14^. Studies performed with cells susceptible to high stretch showed translocation of zyxin to the nucleus upon cell stretching, where it influenced the expression of more than 400 genes mostly related with inhibition of apoptosis and proliferation, reinforcement of ECM interactions and proinflammatory response^15,16^.

In the present study, we aimed at analyzing the significance of the newly identified proteolytic processing events in zyxin to gain further insight how they might influence its complex functions as a major focal adhesion protein in cutaneous wound repair. Using targeted degradomics, we demonstrate proteolytic processing of zyxin at two sites, which we could assign to either caspase-1 or serine protease HtrA1 activity. The zyxin fragment truncated at the HtrA1 site translocated to the nucleus, protected from etoposide-induced cell death, modulated abundance of wound-related proteins in HeLa cells and was endogenously generated at high cell density in primary fibroblasts and in HtrA1-overproducing colon cancer cells. Hence, our results suggest a novel function of HtrA1 in proteolytic control of subcellular localization of zyxin to enhance survival of cells at increased density.

## Materials and methods

### Protein sequence alignment

Amino acid sequence of human and porcine zyxin were aligned using the protein alignment tool from Uniprot (https://www.uniprot.org/align/).

### Culture and transfection of cells

HeLa cells (kind gift from Prof. Dr. C. Azzalin, ETH Zurich, Switzerland) and human dermal fibroblasts (106–05N, Sigma-Aldrich, St. Louis, MI) were cultured in DMEM medium supplemented with 10% fetal bovine serum and 1% (v/v) penicillin/streptomycin in a 5% CO_2_ atmosphere. THP1 monocytes (kind gift from Dr. H.-D. Beer, University of Zurich, Switzerland) and SW480 cells engineered for overproduction or down-regulation of HtrA1^17,18^ (kind gift from Prof. Dr. M. Ehrmann, University Duisburg-Essen, Germany) were cultured in RPMI medium supplemented with 10% fetal bovine serum, 1% (v/v) penicillin/streptomycin plus G418 or puromycin, respectively, in a 5% CO_2_ atmosphere. Transfection was performed using Lipofectamine 2000 according to the manufacturer’s instructions.

### Digestion of cellular extracts with caspase-1

THP1 monocytes were differentiated with 100 nM 12-*O*-tetradecanoylphorbol-13-acetate for 72 h and lysed with bicine buffer (100 mM Bicine, 1% Triton X-100, 250 mM KCl, 1 mM EDTA, 1 mM PMSF, 1 mM 4-(2-aminoethylbenzene) sulfonyl fluoride, and 0.1 mM E-64, pH 8.0). Total cellular extract was treated with human recombinant active caspase-1 (Enzo Life Sciences, Lausen, Switzerland) at a ratio of 0.08 U/μg at 37 °C. The experiment was performed with two different incubation times (2 and 4 h).

### Processing of zyxin by HtrA1

Recombinant human zyxin (Abcam, Cambridge, United Kingdom) was incubated with recombinant human HtrA1 (R&D Systems, Minneapolis, MN) at an enzyme/protein ratio (m/m) of 1/5 for 16 h in 50 mm HEPES (pH 7.8), 100 mM NaCl at 37 °C^19^. Reaction was stopped by heat inactivation and the product was analyzed by immunoblot or PRM. To generate the ‘spike-in control’, an aliquot was digested with GluC and spiked into fibroblast lysates. The experiment was performed in triplicate.

### Immunoblot

Protein samples were resolved by SDS-PAGE and transferred to a nitrocellulose membrane. Unspecific binding sites were blocked with 5% milk, and proteins of interest were detected with antibodies specific for FLAG epitope (Sigma-Aldrich, Buchs, Switzerland), zyxin (B71) (kind gift from Prof. Dr. M. Beckerle, University of Utah, USA), cleaved PARP (Cell Signaling, Danvers, Massachusetts, United States), GAPDH (HyTest, Turku, Finland), matrin-3 (LubioScience GmbH, Zurich, Switzerland), or cleaved caspase-3 (Cell Signaling, Danvers, MA), respectively. Bands were visualized using a horseradish peroxidase conjugated secondary antibody and by exposure of membranes to X-ray films (Fuji Medical, Tokyo, Japan), with a Solo Fusion chemiluminescence recorder (Vilber, Collégien, France) or with a G:BOX chemiluminescence recorder (Syngene, Cambridge, United Kingdom).

### Cloning of zyxin-RFP and zyxin-FLAG

The vector pDsRed-N1 containing full-length zyxin was obtained from Addgene (Plasmid #26720) and used for amplification of N-terminally truncated fragments with primers specific for sequences upstream of the identified cleavage sites. Inserts were cloned into the pDsRed-N1 backbone using HindIII and BamHI cloning sites or into a pFLAG CMV vector (kindly provided by Dr. Tobias Beyer, ETH Zurich, Switzerland) using HindIII and KpnI cloning sites.

**Table:** 

Primer	Sequence
Zyxin^150–572^-RFP	5′ AAA AAA AAG CTT ATG TCT CTG TCC TCA CTG CTG 3′
Zyxin^277–572^-RFP	5′ AAA AAA AAG CTT ATG GCT TCC AAG TTC AGT CCT GGA 3′
Zyxin-RFP reverse	5′ AAA AAA GGA TCC GTC TGG GCT CTA GCA GTG TG 3′
Zyxin-FLAG full-length	5′ AAA AAA GGT ACC ATT ATG GCG GCC CCC C 3′
Zyxin^150–572^-FLAG	5′ AAA AAA GGT ACC ATT ATG GGA TCT CTG TCC TCA CTG CTG 3′
Zyxin^277–572^-FLAG	5′ AAA AAA GGT ACC ATT ATG GCT TCC AAG TTC AGT CCT G 3′
Zyxin-FLAG reverse	5′ AAA AAA AAG CTT GGT CTG GGC TCT AGC 3′

### Fluorescence microscopy of HeLa cells

HeLa cells were grown overnight in chamber slides with 1.7 cm^2^. Cells were transfected as described above and incubated for 48 h. Next, cell culture medium was removed and cells were fixed with 4% PFA for 10 min at RT. Cells were permeabilized with PBS, 0.5% Triton X-100 and blocked with blocking buffer (2% BSA + 0.05% Triton X-100 in PBS). Actin and nuclei were stained by treatment with Phalloidin-Atto 488 (Sigma Aldrich, Buchs, Switzerland) for 1 h and with Hoechst reagent (Sigma Aldrich, Buchs, Switzerland) for 5 min. Slides were mounted with Mowiol/DABCO solution (Sigma Aldrich, Buchs, Switzerland) and visualized using a Zeiss Axio Imager A1 fluorescence microscope equipped with a HXP 120 Laser for fluorophore excitation. The experiments were performed in triplicate.

### Subcellular fractionation of HeLa cells

HeLa cells were grown in culture dishes (ø 6 cm) and lysed in hypotonic lysis buffer (HLB) (10 mM Tris-HCl, pH 7.5, 10 mM NaCl, 3 mM MgCl_2_, 0.3% NP-40) supplemented with c*O*mplete protease inhibitor cocktail (Roche, Basel, Switzerland), 1 mM NaF, and 1 mM Na_3_VO_4_. Lysates were spun down for 10 s at 4 °C and the supernatant was kept as cytoplasmic fraction. The pellet was washed three times with HLB buffer and re-suspended in nuclear lysis buffer (10 mM Tris-HCl (pH 7.5), 0.15 M NaCl, 3 mM MgCl_2_, 0.3% NP-40, 10% glycerol) supplemented with c*O*mplete protease inhibitor cocktail, 1 mM NaF, and 1 mM Na_3_VO_4_. The nuclear extract was sonicated, spun down for 10 s at 4 °C and the supernatant kept as nuclear fraction. The experiments were performed in triplicate.

### Influence of zyxin fragments on apoptosis

HeLa cells were transfected with pFLAG CMV vector as described above. After 48 h, 50 mM etoposide (Sigma-Aldrich, Buchs, Switzerland) was added to culture medium for 2 or 4 h. Cells were scraped off the dish and pelleted by centrifugation (retaining the floating cells), washed with PBS, lysed with Triton X-100 lysis buffer (20 mM Tris-HCl (pH 8.0), 137 mM NaCl, 10% Glycerol, 2 mM EDTA, 1% Triton X-100, c*O*mplete protease inhibitor cocktail) and analyzed by immunoblot. The influence of zyxin on apoptosis was evaluated once in unchallenged cells and once in cells cultured in the presence of etoposide.

### Cell density-dependent proteolysis of zyxin

Human dermal fibroblasts were cultured as above until cellular confluence reached approximately 50%, 80% and for 7 days after full confluence. Growth medium was changed every day and cells were lysed with Triton X-100 lysis buffer before analysis by immunoblot or PRM. The experiments were performed in triplicate.

### Processing of cell extracts for MS analysis

Human dermal fibroblasts and HeLa cells were lysed with Triton X-100 lysis buffer and cell debris was removed by centrifugation. Next, cell extracts were reduced with 3.5 mM TCEP, alkylated with 5 mM chloroacetamide, cleaned by acetone/methanol precipitation and digested for 16 h with trypsin gold (Promega, Madison, WI) or GluC endopeptidase (Roche, Basel, Switzerland).

### Parallel reaction monitoring (PRM)

For PRM analysis of recombinant proteins, digested peptide samples were desalted with custom made C18 stage tips and analyzed with a Q Exactive mass spectrometer (ThermoFisher Scientific, Bremen, Germany) using a 50 cm column at 45 °C. Data was recorded in unscheduled PRM mode over a 70 min gradient with 35000 resolution using 1e6 ions with isolation windows of 1.2 m/z and 500 ms injection time. For PRM analysis of fibroblast cellular extracts, samples were analyzed with a Q Exactive HF-X instrument (ThermoFisher Scientific, Bremen, Germany) using a 50 cm column at 45 °C. Data was recorded in unscheduled PRM mode over a 70 min gradient with 30000 resolution using 3e6 ions with isolation windows of 1.2 m/z. Maximum injection times of 700 ms and 100 ms were set for analysis of GluC generated zyxin cleavage and GAPDH peptides, respectively. For detection of tryptic peptides, 5e5 ions were used with injection times of 200 ms.

### Tandem mass tags (TMT)-based proteomics

HeLa cells were transfected in triplicates with pFLAG CMV vector encoding full length zyxin and zyxin^277–572^ and cultured for 48 h before lysis with Triton X-100 lysis buffer as described above. Peptides were labeled with TMT reagents (ThermoFisher Scientific, Rochford, IL) according to the manufacturer’s instructions. Briefly, 50 µg digested peptides per condition were labeled at a 4/1 (w/w) TMT/protein ratio for 1 h at room temperature. Labeling reaction was quenched with 100 mM ammonium bicarbonate for 30 min. Next, peptide samples were mixed, desalted with sep-pak C18 columns (Waters, Milford, MA) and analyzed on a Q Exactive (ThermoFisher Scientific, Bremen, Germany) instrument using a 50 cm column at 45 °C. Data was recorded in data-dependent acquisition mode over a 140 min gradient using full-MS scans recorded with 70000 resolution using 3e6 ions for MS1, and resolution 35000, top 10 precursors with isolation windows of 1.6 m/z and 120 ms transient time for MS2.

### MS data interpretation

Raw data files were searched using Sequest from ProteomeDiscoverer 2.2 (ThermoFisher Scientific, Waltham, MA) against a database compiled from the UniProt reference proteome for Homo Sapiens (taxid: 9606, v2017–10–25) with the following parameters: full trypsin enzyme specificity allowing up to 1 missed cleavage; carbamidomethyl(C), TMT-6plex (N-term) and TMT-6plex (K) as fixed modifications; acetyl (N-term), pyroQ (N-term), oxidation (M), deamidation (NQ) as variable modifications; parent mass error at 10 ppm, fragment mass error at 0.02 Da.

### Normalization, annotation and statistical analysis

For normalization of TMT-channels, the function “normalize to peptide amount” was selected in ProteomeDiscoverer 2.2. Next, the value of each channel was normalized to the pooled reference channel. Subsequently pairwise comparisons with two-sided *t*-tests were performed using Microsoft Excel. Raw *p* < 0.05 was considered significant. Heatmaps of protein abundances were created using MeV Viewer (www.tm4.org).

## Results

### Zyxin is proteolytically processed in cutaneous wound exudates

**Fig. 1:**
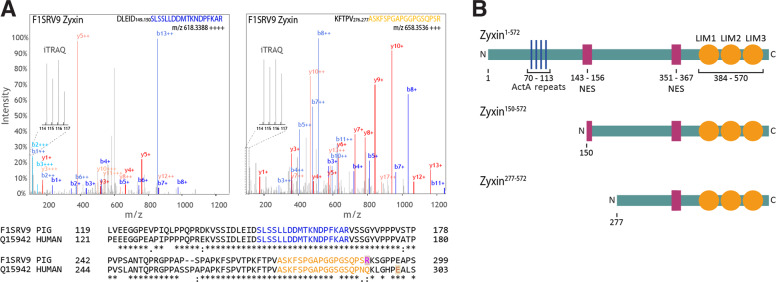
Schematic representation of zyxin truncations identified in porcine wound exudates. **a** Representative spectra of identified neo-N termini and mapping to porcine and human zyxin. Data extracted from Sabino et al.^6^. Blue and orange sequences represent the identified peptides. ‘iTRAQ’ reporter ions indicate N-terminal labeling. Purple ‘R’ represents the C-terminal arginine present in the porcine peptide. Brown ‘E’ shows the glutamate exploited as GluC cleavage site for peptide identification in samples from human origin. **b** Scheme of full length zyxin and N-terminal truncations identified in porcine wound exudates. Representation of zyxin domains according to Smith et al.^7^.

In a recent study, we systematically assessed limited proteolysis in the healing skin wound in vivo by quantitative degradomics analysis of wound exudates from a porcine wound model^6^. From this dataset we identified a novel proteolytic cleavage of zyxin C-terminal to Val276 as well as a known processing site C-terminal to Asp149 that had been previously associated with caspase-1 activity (Fig. 1a)^20^. Importantly, both cleavage site sequences are conserved between pig and human (Fig. 1a), suggesting that the proteolytic processing of zyxin might influence its biological function. Concomitant identification of multiple internal tryptic peptides C-terminal to cleavage sites (Supplementary Fig. S1) indicated generation of stable N-terminally truncated zyxin fragments comprising its LIM domains, and C-terminal nuclear export sequence (Fig. 1b). High coverage of this part of the protein has also been observed by others in pig and human tissues as well as in human plasma^21,22^.

### Caspase-1 and serine protease HtrA1 are responsible for zyxin cleavage

**Fig. 2:**
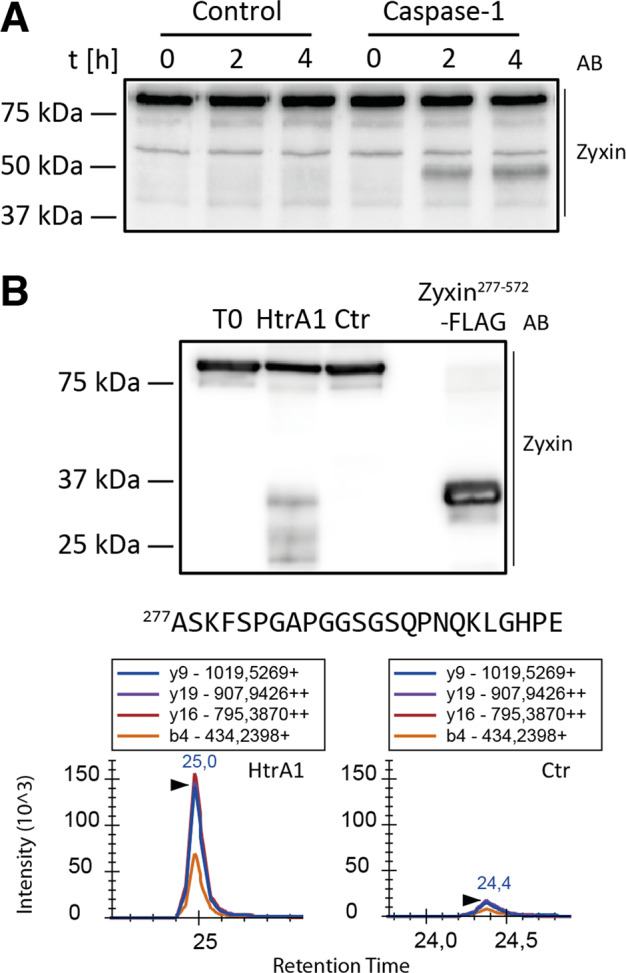
Processing of zyxin by caspase-1 and serine protease HtrA1. **a** Proteolytic processing of zyxin by caspase-1. Cellular lysates from differentiated THP1 monocytes were incubated with recombinant caspase-1 for 2 or 4 h and analyzed by immunoblot with an antibody raised against the C-terminal region of zyxin (B71). **b** Proteolytic processing of zyxin by HtrA1. Recombinant human zyxin was incubated with recombinant human HtrA1 for 16 h and analyzed by immunoblot (as in **a**) and by parallel reaction monitoring (PRM). PRM assay was based on a semi-GluC peptide released from human zyxin (Fig. 1A). Cellular lysates from HeLa cells expressing the fragment zyxin^277–572^-FLAG were used in the immunoblot analysis for molecular weight comparison.

Identification of the protease responsible for a particular proteolytic event is essential for understanding the influence of protein processing in physiological contexts. Therefore, we treated cellular extracts from differentiated THP1 monocytes with recombinant caspase-1 and by immunoblot analysis observed a fragment indicating cleavage C-terminal to Asp149 and confirming previous results from Agard et al.^20^ (Fig. 2a). To identify the protease responsible for processing of zyxin C-terminal to Val276, we focused on proteases concomitantly identified with the respective zyxin processing in wound exudates^6^ and mined the literature and bioinformatics resources (MEROPS^23^, TopFind^24^) for proteases with preference for amino acid sequences that resemble the identified cleavage site (Fig. 1a). We selected serine protease HtrA1 (HtrA1) as a candidate protease, since HtrA1 was also highly abundant in wound exudates^6^ and recently described to prefer small hydrophobic residues (such as valine) in the P1 position^19^. To assess the capacity of HtrA1 to process zyxin, we incubated recombinant human zyxin with recombinant human HtrA1 and analyzed the cleavage product by immunoblot with an antibody directed against the C-terminal region of zyxin (Fig. 2b). Thereby, we observed generation of a fragment with expected molecular weight (~37 kDa), confirming the ability of HtrA1 to cleave zyxin. Next, since immunoblot analysis does not provide sequence information of the cleavage site, we developed a parallel reaction monitoring (PRM)-based targeted degradomics assay^25^ to evaluate the generation of the human homolog of the respective zyxin neo-N terminus identified in vivo. Applying this PRM assay to recombinant human zyxin incubated with recombinant human HtrA1, we identified the expected neo-N-terminal peptide with an intensity approximately one order of magnitude higher in the HtrA1 treated than the control sample (Fig. 2b). Hence, our data identified HtrA1 as a candidate protease cleaving zyxin C-terminal to Val276 during cutaneous wound repair.

### Processing of zyxin at the HtrA1 cleavage site generates a fragment that translocates to the nucleus

**Fig. 3:**
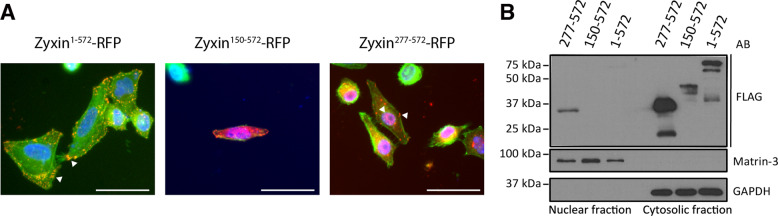
Subcellular localization of proteolytic fragments of zyxin. **a** Representation of zyxin-RFP subcellular localization. HeLa cells were transfected with RFP fusion constructs of full-length zyxin and truncated fragments. Forty-eight hours after transfection cell nuclei were stained with Hoechst, actin was stained with Phalloidin-Atto 488, and localization of zyxin was observed by fluorescence microscopy. Scale bar: 50 µm. **b** Immunoblot analysis of subcellular fractions of HeLa protein extracts. HeLa cells were transfected with FLAG-tagged full-length zyxin and truncated fragments. Forty-eight hours after transfection cells were fractionated into cytosolic and nuclear fractions and analyzed by immunoblot using an antibody directed against FLAG epitope. GAPDH (cytosolic protein) and matrin-3 (nuclear protein) were used as controls for efficiency of subcellular fractionation.

Since zyxin can shuttle between focal adhesions and the nucleus^11^, we evaluated the influence of N-terminal truncation of zyxin on its subcellular distribution. For this purpose, zyxin-RFP and zyxin-FLAG fusion constructs corresponding to full length zyxin and the two N-terminally truncated fragments were expressed in cultured HeLa cells. As expected^26^, visualization of RFP signal revealed localization of full-length zyxin to focal adhesions. The fragment zyxin^277–572^ readily translocated to the nucleus, whereas cells expressing zyxin^150–572^ presented a globally low RFP signal and few RFP positive cells (Fig. 3a). Moreover, the few RFP positive cells showed morphological signs of dying cells. Immunoblot analysis of cytosolic and nuclear subcellular fractions of HeLa cells expressing zyxin-FLAG validated localization of full-length zyxin in the cytoplasm, presence of zyxin^277–572^ in the nucleus and lower expression levels of zyxin^150–572^, confirming the observations made by fluorescence microscopy (Fig. 3b). Additionally, the immunoblot showed fragmentation of full-length zyxin with formation of two fragments of molecular weights comparable with the truncated versions, supporting intracellular and specific proteolysis, potentially by caspase-1 and HtrA1.

### Cell density determines proteolytic processing of endogenous zyxin at the HtrA1 cleavage site

**Fig. 4:**
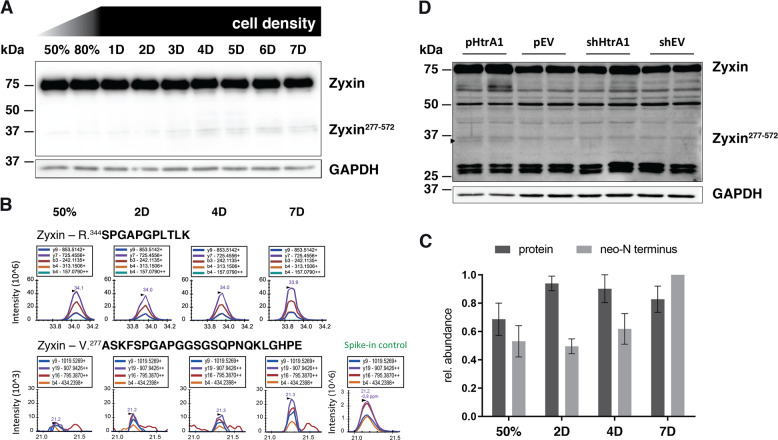
Endogenous and cell density-dependent proteolysis of zyxin. Human dermal fibroblasts were cultured until reaching a cellular density of approximately 50%, 80% and for 7 days after reaching confluency. **a** Immunoblot analysis of zyxin fragmentation at distinct cell densities with an antibody directed against the C-terminus of zyxin (B71). **b** Analysis of zyxin semi-GluC neo-N terminus and fully tryptic peptides (see also Supplementary Fig. S2) in cellular extracts of fibroblasts cultured at distinct cellular densities. Fully tryptic and GluC peptides of GAPDH were monitored in each MS run for loading control (see also Supplementary Figs. S3 and S4). ‘Spike-in control’ was generated by incubating recombinant human zyxin with HtrA1 and GluC and spiking into fibroblast extracts. **c** Relative abundances of zyxin ^277^neo-N terminus and total protein in lysates from fibroblasts grown to increasing cell densities measured by PRM. Data were recorded in three independent experiments, normalized to GAPDH and scaled to the maximum (for PRM traces, see Supplementary Figs. S5 to S10). Error bars: SD. **d** Immunoblot analysis of zyxin fragmentation in HtrA1-overproducing (pHtrA1) and HtrA1-silenced (shHtrA1) SW480 cells and vector-transfected controls (pEV, shEV)^17,18^. Black arrow points to band at ~37 kDa only present in samples from HtrA1-overproducing cells. Cells were analyzed in duplicates.

Interestingly, we observed that the abundance of the zyxin fragment with a molecular weight of ~37 kDa was dependent on the number of days cells had been cultured between transfection with the full-length construct and lysis. Hence, to test the hypothesis that high cell density induces proteolytic processing of endogenous zyxin, we cultured human dermal fibroblasts for seven days after cells reached 100% confluency and assessed the fragmentation of zyxin by immunoblot. Indeed, endogenous zyxin was increasingly processed in cells cultured for longer periods (Fig. 4a). To correlate the observed fragment with the truncation generated by HtrA1, we used PRM to quantify the neo-N terminus starting at A277 and the abundance of zyxin in cells cultured for distinct periods. We observed an increase in abundance of the neo-N terminus potentially generated by HtrA1 from day 4 to day 7, while the zyxin protein only increased in abundance from 50% confluency to day 2 but showed no change from day 4 to day 7 (Fig. 4b, c; Supplementary Figs. S2–S10), indicating regulated limited proteolysis of zyxin during increase in cell density. In addition, a fragment of the same size was observed in SW480 cells genetically engineered for overproduction of HtrA1 but not in controls, indicating that indeed HtrA1 activity is responsible for this cleavage and not another protease with closely related specificity (Fig. 4d).

### Nuclear HtrA1-generated zyxin fragment has anti-apoptotic properties

**Fig. 5:**
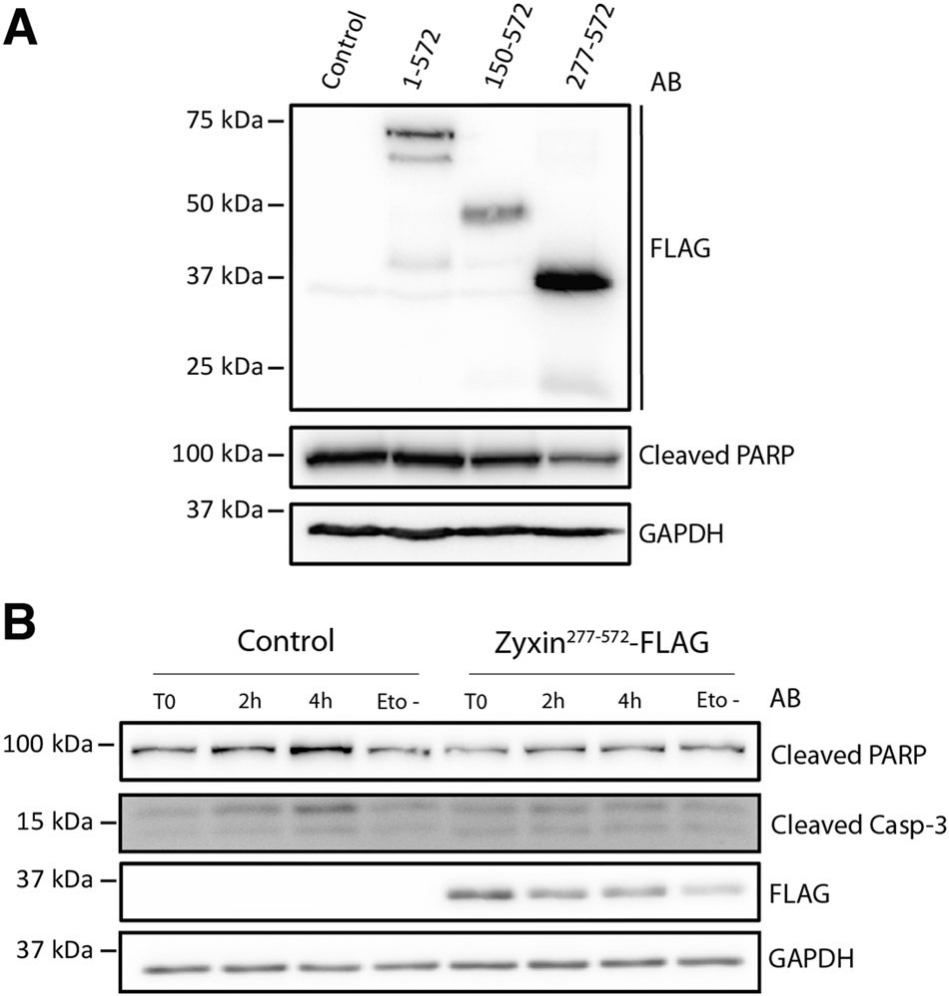
Effect of ectopic expression of zyxin on apoptosis. **a** Effect of expression of zyxin-FLAG on levels of cleaved PARP. HeLa cells were transfected with zyxin-FLAG fusion constructs. Forty-eight hours post transfection cells were lysed and analyzed by immunoblot with an antibody raised against cleaved PARP. **b** Effect of expression of zyxin^277–572^ in cells under apoptotic stress. Cultured HeLa cells were transfected with zyxin^277-57^-FLAG fusion construct and 48 h after transfection treated with the apoptosis inducer etoposide. The activation of caspase-3 and cleavage of PARP was monitored by immunoblot.

Upon transient transfection, HeLa cells expressed significantly higher levels of zyxin^277–572^ than of full length zyxin or zyxin^150–572^ (Fig. 3b). Previous studies described pro- or anti-apoptotic properties of zyxin, depending on tissue, cell type and stimulus^27–30^. To test the hypothesis that the differences in expression of full-length zyxin and the two truncated fragments resulted from pro- or anti-apoptotic functions of the ectopically expressed fragments, we transfected HeLa cells with zyxin-FLAG fusion constructs and monitored levels of cleaved PARP^31^ 48 h after transfection. Interestingly, we did not observe differences in the levels of cleaved PARP between control, full-length zyxin and zyxin^150–572^, whereas cells transfected with zyxin^277–572^ showed lower intensity of the PARP cleavage fragment (Fig. 5a). To further test the anti-apoptotic properties of HtrA1-truncated zyxin, we treated HeLa cells expressing zyxin^277–572^ with etoposide and monitored activation of caspase-3 and PARP cleavage by immunoblot. Thereby, we observed a lower caspase-3 activation and PARP processing in cells expressing zyxin^277–572^ than in control cells (Fig. 5b), confirming the pro-survival properties of zyxin^277–572^. Hence, our data suggest that the lower expression of zyxin^150–572^ is most likely due to degradation of the fragment, while the higher expression of zyxin^277–572^ is most likely associated with pro-survival properties of the fragment.

### Ectopic expression of zyxin^277–572^ modulates abundance of wound-related proteins

**Fig. 6:**
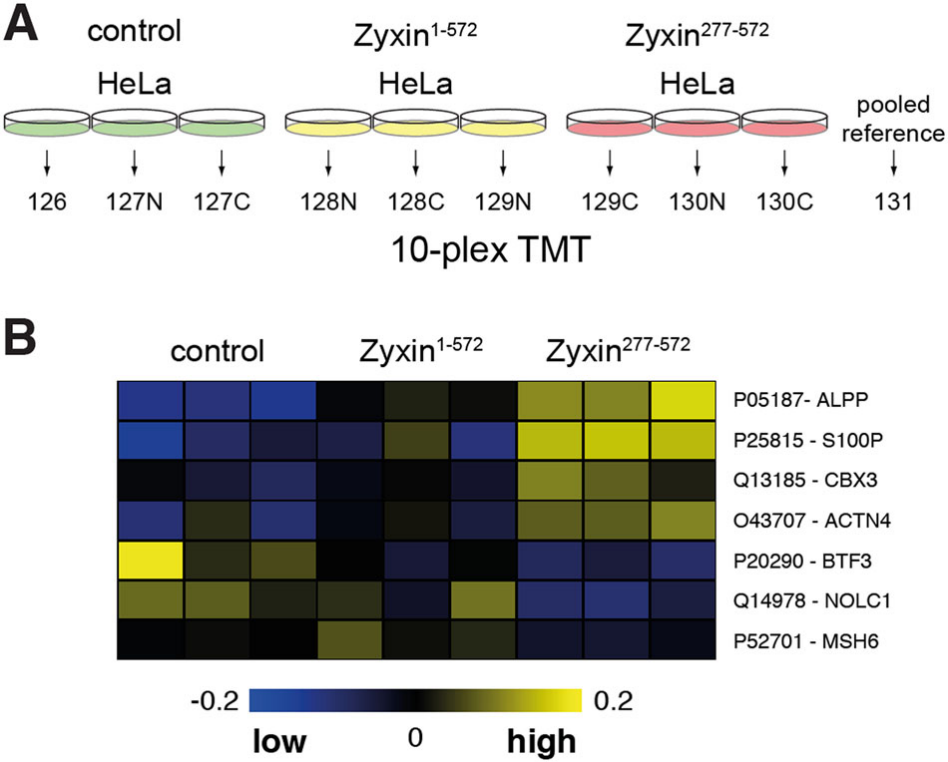
Tandem mass tags (TMT)-based proteome quantification of HeLa cells expressing full length zyxin and zyxin^277–572^. **a** Experimental design for quantitative proteomics analysis of HeLa cells expressing zyxin-FLAG fusion constructs. HeLa cells were transfected with full length zyxin, zyxin^277–572^ or mock transfected in triplicates. Forty-height hours post transfection cells were lysed, tryptic protein digests labeled with TMT isobaric tags and analyzed by MS using a pooled reference for experimental normalization. **b** Subset of proteins with significant differences in abundance between cells expressing zyxin^277–572^ and full length zyxin and mock control group (raw *p*-value<0.05). The proteins alkaline phosphatase (ALP), protein S100-P, heterochromatin protein 1 homolog gamma (HP1γ) and α-actinin-4 (ACTN4) showed higher abundance in cells expressing zyxin^277–572^, while the proteins basic transcription factor 3 (BTF3), nucleolar and coiled-body phosphoprotein 1 (NOLC1) and DNA mismatch repair protein MSH6 showed lower abundance in cells expressing zyxin^277–572^. Heatmap representation of protein abundances was produced in MeV Viewer (www.tm4.org). Values were mean-centered and color scale limits are indicated.

To elucidate the molecular mechanism underlying the pro-survival phenotype observed in cells expressing zyxin^277–572^, we transiently expressed full-length zyxin-FLAG and zyxin^277–572^-FLAG fusion constructs in cultured HeLa cells and analyzed the quantitative changes in cellular proteomes by tandem mass tag (TMT)-based quantitative proteomics (Fig. 6a; Supplementary Table S1). For statistically robust comparison of distinct conditions, we exploited the multiplexing capabilities of TMT isobaric tags and analyzed three replicates per condition within the same experiment, using a pooled reference for scaling. By student t-tests we performed pairwise comparisons of protein abundance in cells expressing zyxin^277–572^ with full-length zyxin and mock transfection (control). Thereby, we identified a subset of four proteins (alkaline phosphatase (ALP), protein S100-P, heterochromatin protein 1 homolog gamma (HP1γ) and α-actinin-4 (ACTN4)) with significantly higher abundance in cells expressing zyxin^277–572^ and a subset of three proteins (basic transcription factor 3 (BTF3), nucleolar and coiled-body phosphoprotein 1 (NOLC1) and DNA mismatch repair protein MSH6) with lower abundance in the same condition (Fig. 6b; Supplementary Table S2) (raw p-value<0.05). These results suggest that nuclear zyxin^277–572^ may regulate expression of proteins related to cell proliferation and migration (S100P, ACTN4 and BTF3), protein synthesis (CBX3, BTF3, NOLC1) and genome stability (CBX3, BTF3, NOLC1 and MSH6), processes which are all related to wound healing progression.

## Discussion

**Fig. 7:**
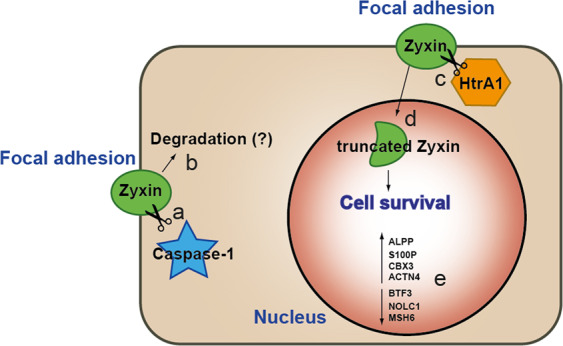
Proposed model for the biological consequences of zyxin proteolysis. Proteolysis of zyxin by caspase-1 C-terminal to Asp149 generates a C-terminal fragment that is most likely degraded (**a**). High cellular density activates HtrA1 (**b**) which processes zyxin C-terminal to Val276 (**c**) and generates a C-terminal fragment that migrates to the nucleus (**d**). Nuclear zyxin^277–572^ induces cell survival potentially by increasing the expression of ALP, HP1γ, ACTN4 and S100-P and reducing the expression of BTF3, NOLC1 and MSH6 (**e**).

In this study, we characterized the biological relevance of two proteolytic cleavages of the focal adhesion protein zyxin, which we had identified in vivo by iTRAQ-TAILS analysis of porcine wound exudates^6^. A cleavage event C-terminal to Asp149 was previously associated with caspase-1 activity^20^, yet the biological relevance has not been assessed. Here, we provide evidence that a C-terminal zyxin fragment related to processing by caspase-1 can only be expressed with low efficiency in mammalian cells, suggesting instability of the truncated protein. Moreover, we describe for the first time a proteolytic mechanism regulating zyxin’s subcellular localization by inducing its migration to the nucleus. We propose that this nuclear translocation is induced by HtrA1-mediated proteolysis of zyxin C-terminal to Val276 in response to cues triggered by cell density and that nuclear zyxin^277–572^ affects cell survival and homeostasis in part by increasing expression of ALPP, CBX3, ACTN4 and S100P and reducing expression of BTF3, NOLC1 and MSH6. These observations provide novel insight into limited proteolysis as a signaling mechanism transmitting information from the cell periphery to the nucleus under conditions of altered cellular environments in response to environmental stress (Fig. 7).

Agard *et al*. identified caspase-1-dependent processing of zyxin C-terminal to Asp149 both in vitro by incubation of lysates with the active protease and in cells in response to several inflammatory stimuli^20^. Having identified this cleavage in liquid biopsies from wounds and thus samples from a complex inflammatory tissue response^6^ indicates for the first time its in vivo relevance. In vitro efficiency of zyxin cleavage by caspase-1 was very low^20^, which would be in agreement with our findings that forced expression of the related C-terminal fragment generates an unstable truncated protein with detrimental effects on cell viability. This warrants further investigations to understand the fine-tuned regulation of this cleavage event and how it contributes to cellular responses under inflammatory stress conditions in various cell types.

In an effort to elucidate the importance of different regions of zyxin for its subcellular localization, Nix *et al*. expressed truncated chicken zyxin-GFP (c-zyxin) fusion constructs in cultured REF52 cells^13^. The authors detected c-zyxin^131–542^ in focal adhesions (corresponding to human zyxin^123–572^), c-zyxin^245–542^ (human zyxin^288–572^) in focal adhesions and c-zyxin^305–572^ (human zyxin^340–572^) in focal adhesions and the nucleus. Our observation of nuclear accumulation of zyxin^277–572^ suggests that the 143-156 NES (Fig. 1b) plays an important role in nuclear export of zyxin, and that the 351-366 NES is not sufficient for nuclear export of the protein. Deletion of the 322-331 NES (homologous of 351-366 NES in human zyxin) promotes nuclear accumulation of chicken zyxin^13^, but chicken zyxin lacks the NES consensus sequence closest to the N terminus^32^. Interestingly, based on NetNES prediction^33^ pig and mouse zyxin also harbor both NES sequences homologs to the human sequence. This might indicate evolution towards multiple NESs to ensure increased flexibility in control of subcellular localization. Importantly, to our knowledge, our study provides the first evidence of a proteolytic mechanism that induces translocation of zyxin to the nucleus.

With HtrA1, we succeeded to identify a protease that is capable of directly processing zyxin at the observed cleavage site C-terminal to Val276. HtrA1 is predominantly secreted, but at least 20% of the HtrA1 pool is localized in the cytoplasm^34^. Intracellularly, it can be associated with microtubules and proteolytically process components of the cytoskeleton to regulate cell motility and alter mechanical cell phenotypes^35,36^. Moreover, HtrA1 degrades unfolded and misfolded proteins, thereby reducing the burden of unfolded proteins, contributing to proteostasis and cell survival^34,37,38^. Thus, there is clear evidence for intracellular HtrA1-mediated proteolysis, which is further supported by this study but needs more exploration to better understand the pleiotropic activities of this evolutionary conserved protease.

Translocation to the nucleus has been observed for zyxin in response to stretch-induction in endothelial cells as it appears in vasoconstriction^15,16^. Wound fibroblasts also experience mechanical tension^39^ but an overall distinct extracellular environment, which might elicit different cellular responses. Here, limited proteolysis rather than cytoskeletal rearrangement might be used to disrupt zyxin’s attachment to focal adhesions, facilitate nuclear translocation and regulation of gene transcription. Zyin^277–572^, the zyxin fragment corresponding to HtrA1 cleavage, was endogenously generated in fibroblasts at high cell density (Fig. 4). Increased fibroblast density is also seen in wound dermis^40^ and might explain presence of zyxin^277–572^ in wound exudate sampled at the onset of granulation tissue formation^6^, but the definite environmental cue to trigger zyxin cleavage has still to be discovered.

After generation by HtrA1 zyxin^277–572^ retains its LIM domains, which have been shown to be able to either directly interact with DNA or other proteins to mediate zyxin’s activities in regulating gene expression^41^. Overexpression of zyxin^277–572^ in HeLa cells moderately but significantly regulated abundances of proteins that have been associated with maintaining cellular homeostasis in environments of rapid proliferation and migration (Fig. 6)^42–48^. This is in agreement with observations that zyxin influences cell migration, adhesion and proliferation as well as its potential involvement in cancer progression^49–53^. Zyxin’s role in regulating cell survival is controversial^27–30^, but nuclear activities have generally been related to anti-apoptotic effects^15,16^. This is also supported by our own findings, which directly correlated high expression of nuclear zyxin^277–572^ with reduced abundance of apoptosis markers. Thus, induced nuclear translocation of zyxin appears as protective response in conditions requiring extended cell viability like in tissue repair.

In the healing skin wound, keratinocytes and fibroblasts undergo a series of complex changes leading to a hyperproliferative and migratory phenotype transiently resembling cancer cells^54^. Our newly identified HtrA1-zyxin proteolytic signaling pathway presents a mechanism to support this phenotype by contributing to enhanced cell survival under conditions of high cell density. Hence, regulation of expression and activity of the HtrA1 protease might be exploited to advance granulation tissue formation in non-healing wounds^4^.

## Supplementary information

Supplementary Figure Legends

Figure S1

Figure S2

Figure S3

Figure S4

Figure S5

Figure S6

Figure S7

Figure S8

Figure S9

Figure S10

Supplementary Tables S1 and S2

Fibroblasts_GluC_digestion_replicate1.sky

Fibroblasts_GluC_digestion_replicates_2_3.sky

Fibroblasts_Trypsin_digestion_replicate1.sky

Fibroblasts_Trypsin_digestion_replicates_2_3.sky

Recombinant_zyxin_HtrA1.sky

## Data Availability

Data for PRM analysis are available as Skyline zip archive in Supplemental Material. The mass spectrometry proteomics data have been deposited to the ProteomeXchange Consortium (http://proteomecentral.proteomexchange.org)^55^ via the PRIDE partner repository^56^ with the dataset identifier PXD017432.
