# Supplementary material for: Cell density-dependent proteolysis by HtrA1 induces translocation of zyxin to the nucleus and increased cell survival
Source: Cell Death Dis. 2020 Aug 21;11(8):674. doi: 10.1038/s41419-020-02883-2 (PMC7442833; doi:10.1038/s41419-020-02883-2)
Supplement: Supplementary file 1 — Supplementary Figure Legends [file 41419_2020_2883_MOESM1_ESM.docx]

**Figure S1:** Positional proteomics analysis of zyxin in pig wound exudates. FASTA sequence of zyxin from UniProt (release 2013_01). Peptides identified with high confidence by iTRAQ-TAILS in pig wound exudates are indicated in red and blue. Semi-tryptic peptides have been identified as protein N-termini, fully tryptic peptides as internal peptides. Note that in iTRAQ-TAILS lysines are labeled and thus skipped by trypsin cleavage. #PSM indicates number of peptide-to-spectrum matches for each peptide in the referenced dataset. Ac: natural acetylation; iTRAQ: N-terminal label

Data have been extracted from:

Sabino F, Egli FE, Savickas S, Holstein J, Kaspar D, Rollmann M, Kizhakkedathu JN, Pohlemann T, Smola H, auf dem Keller U. Comparative Degradomics of Porcine and Human Wound Exudates Unravels Biomarker Candidates for Assessment of Wound Healing Progression in Trauma Patients. *J Invest Dermatol.* 2018 Feb;138(2):413-422. doi: 10.1016/j.jid.2017.08.032. Epub 2017 Sep 9. PMID: 28899681.

ProteomeXchange: PXD006674

**Figure S2:** PRM analysis of tryptic peptides of zyxin in cellular extracts from dermal fibroblasts cultured for distinct periods. Cultured cells were lysed at approximately 50% confluency and 2, 4, and 7 days after reaching confluency. Transitions of fragment ions were monitored for identification of the precursors FSPGAPGGSGSQPNQK, QHPVPPPAQNQNQVR and SPGAPGPLTLK.

**Figure S3:** PRM analysis of tryptic peptides of GAPDH in cellular extracts from dermal fibroblasts cultured for distinct periods. Cultured cells were lysed at approximately 50% confluency and 2, 4, and 7 days after reaching full confluency. Transitions of fragment ions were monitored for identification of the precursors LISWYDNEFGYSNR, LVINGNPITIFQER and VGVNGFGR.

**Figure S4:** PRM analysis of GluC-generated peptides of GAPDH in cellular extracts from dermal fibroblasts cultured for distinct periods. Cultured cells were lysed at approximately 50% confluency and 2, 4, and 7 days after reaching full confluency. Transitions of fragment ions were monitored for identification of the precursors FGYSNRVVD, GPLKGILGYTE and STGVFTTME.

**Figure S5:** PRM analysis of zyxin semi-GluC neo-N terminus and tryptic peptides in cellular extracts from dermal fibroblasts cultured for distinct periods (Replicate 2). Cultured cells were lysed at approximately 50% confluency and 2, 4, and 7 days after reaching confluency. Transitions of fragment ions were monitored for identification of the precursors ASKFSPGAPGGSGSQPNQKLGHPE (GluC digest), FSPGAPGGSGSQPNQK QHPVPPPAQNQNQVR and SPGAPGPLTLK (trypsin digest). ‘Spike-in control’ was generated by incubating recombinant human zyxin with HtrA1 and GluC and spiking into fibroblast extracts.

**Figure S6:** PRM analysis of tryptic peptides of GAPDH in cellular extracts from dermal fibroblasts cultured for distinct periods (Repliacte 2). Cultured cells were lysed at approximately 50% confluency and 2, 4, and 7 days after reaching full confluency. Transitions of fragment ions were monitored for identification of the precursors LISWYDNEFGYSNR, LVINGNPITIFQER and VGVNGFGR.

**Figure S7:** PRM analysis of GluC-generated peptides of GAPDH in cellular extracts from dermal fibroblasts cultured for distinct periods (Replicate 2). Cultured cells were lysed at approximately 50% confluency and 2, 4, and 7 days after reaching full confluency. Transitions of fragment ions were monitored for identification of the precursors FGYSNRVVD, GPLKGILGYTE and STGVFTTME.

**Figure S8:** PRM analysis of zyxin semi-GluC neo-N terminus and tryptic peptides in cellular extracts from dermal fibroblasts cultured for distinct periods (Replicate 3). Cultured cells were lysed at approximately 50% confluency and 2, 4, and 7 days after reaching confluency. Transitions of fragment ions were monitored for identification of the precursors ASKFSPGAPGGSGSQPNQKLGHPE (GluC digest), FSPGAPGGSGSQPNQK QHPVPPPAQ-

NQNQVR and SPGAPGPLTLK (trypsin digest). ‘Spike-in control’ was generated by incubating recombinant human zyxin with HtrA1 and GluC and spiking into fibroblast extracts.

**Figure S9:** PRM analysis of tryptic peptides of GAPDH in cellular extracts from dermal fibroblasts cultured for distinct periods (Replicate 3). Cultured cells were lysed at approximately 50% confluency and 2, 4, and 7 days after reaching full confluency. Transitions of fragment ions were monitored for identification of the precursors LISWYDNEFGYSNR, LVINGNPITIFQER and VGVNGFGR.

**Figure S10:** PRM analysis of GluC-generated peptides of GAPDH in cellular extracts from dermal fibroblasts cultured for distinct periods (Replicate 3). Cultured cells were lysed at approximately 50% confluency and 2, 4, and 7 days after reaching full confluency. Transitions of fragment ions were monitored for identification of the precursors FGYSNRVVD, GPLKGILGYTE and STGVFTTME.
