# Supplementary figures and images for: Cell density-dependent proteolysis by HtrA1 induces translocation of zyxin to the nucleus and increased cell survival
Source: Cell Death Dis. 2020 Aug 21;11(8):674. doi: 10.1038/s41419-020-02883-2 (PMC7442833; doi:10.1038/s41419-020-02883-2)

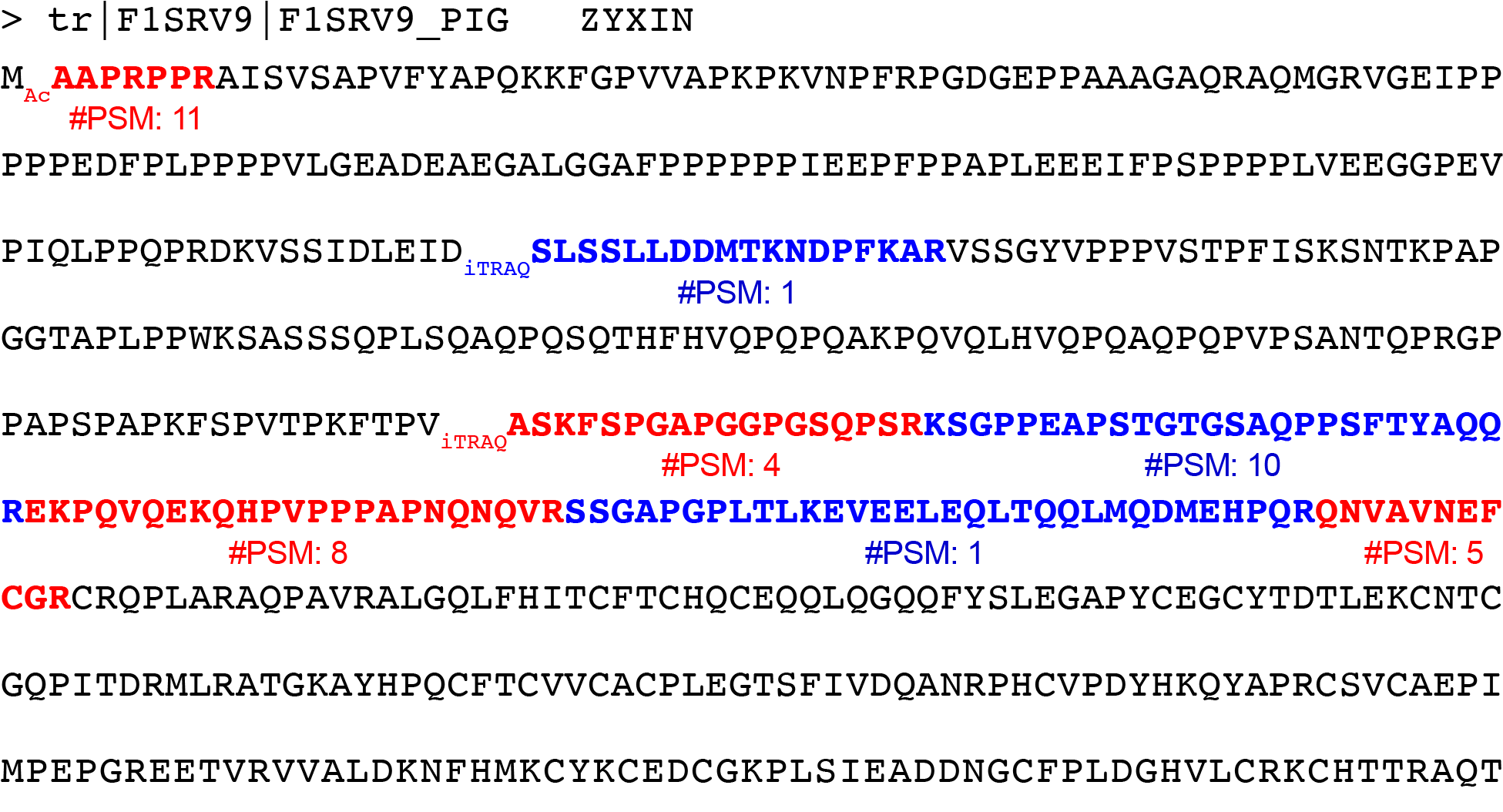

Supplement: Supplementary file 2 — Figure S1 [file 41419_2020_2883_MOESM2_ESM.png]

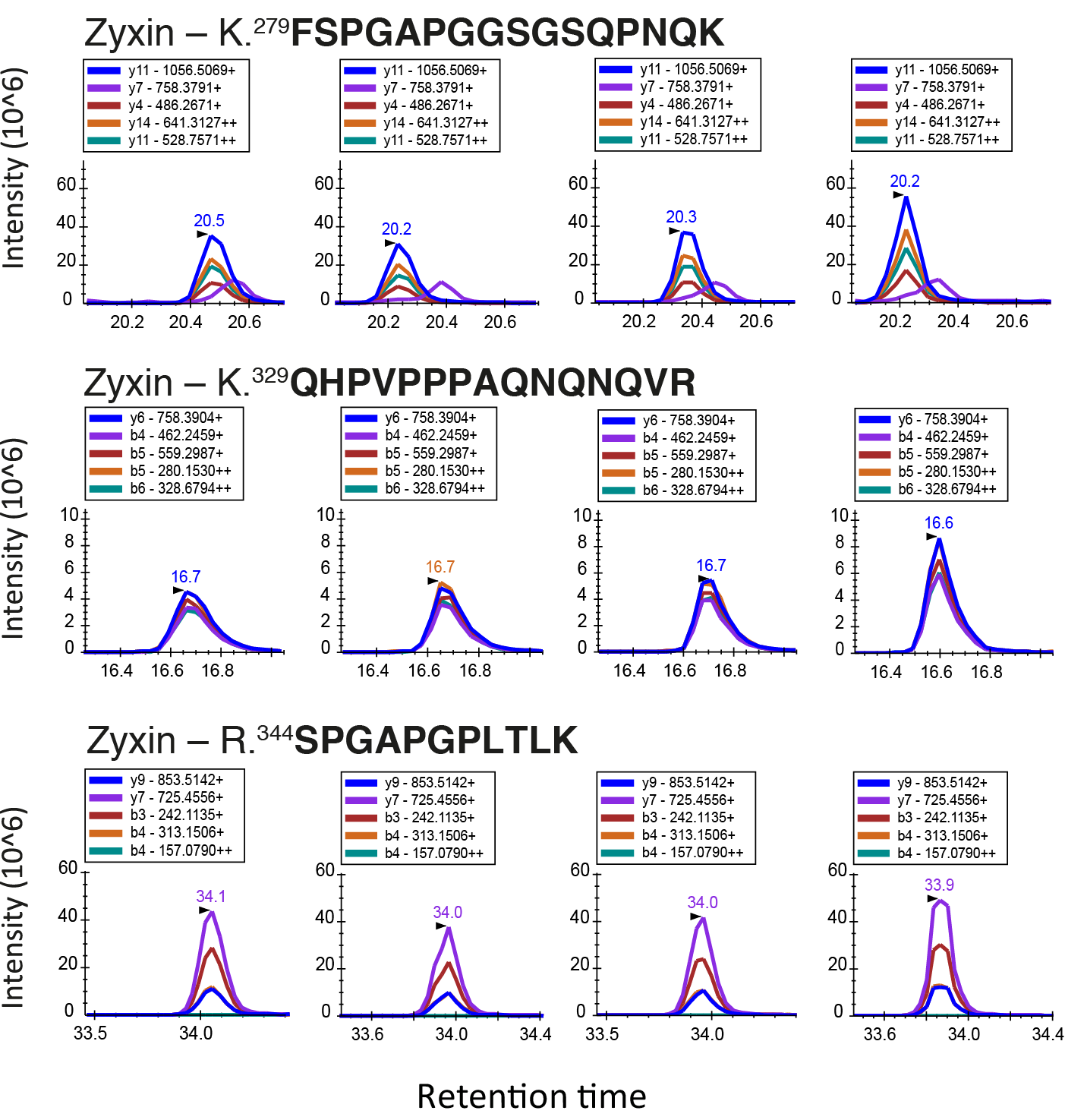

Supplement: Supplementary file 3 — Figure S2 [file 41419_2020_2883_MOESM3_ESM.png]

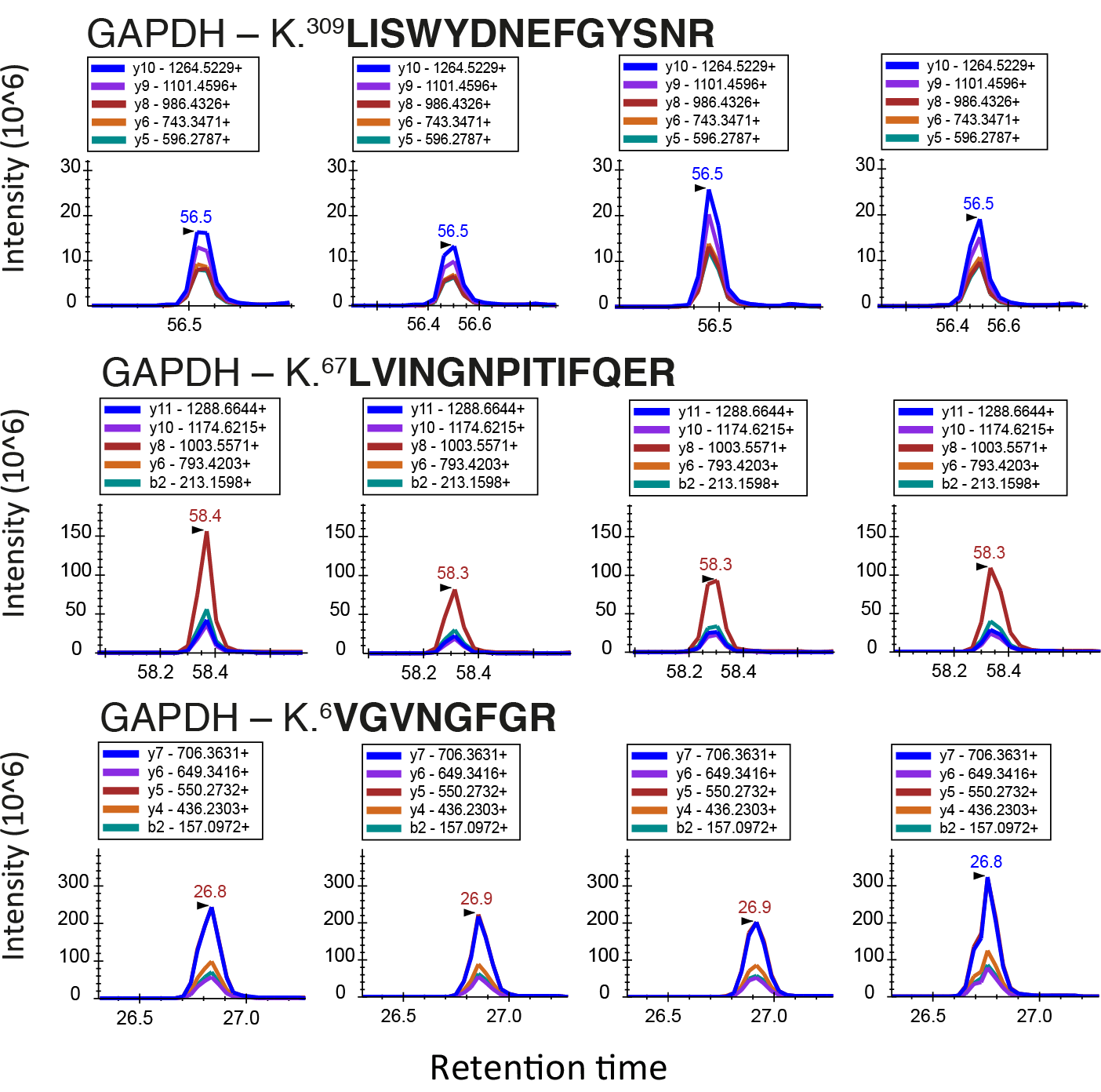

Supplement: Supplementary file 4 — Figure S3 [file 41419_2020_2883_MOESM4_ESM.png]

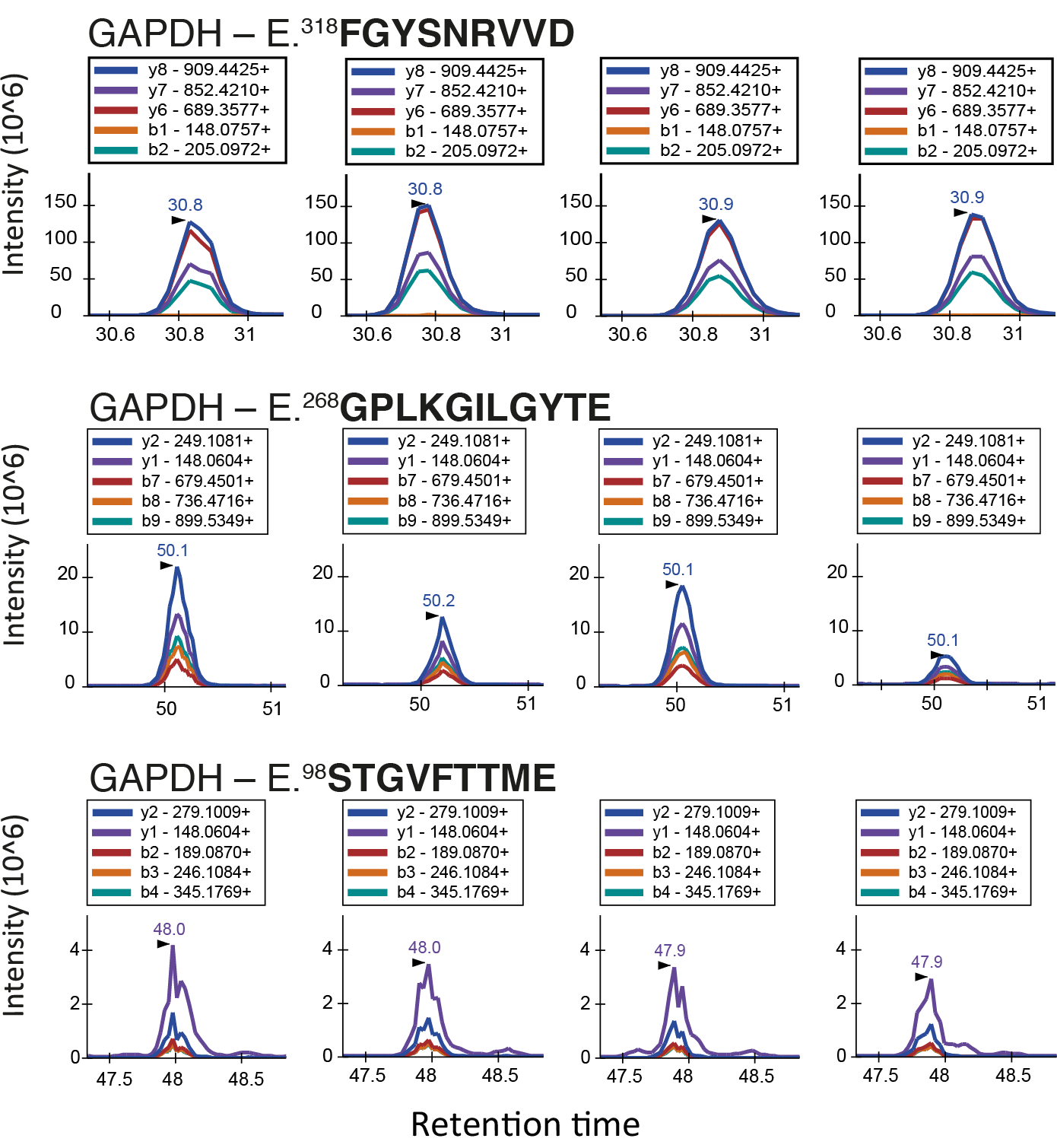

Supplement: Supplementary file 5 — Figure S4 [file 41419_2020_2883_MOESM5_ESM.png]

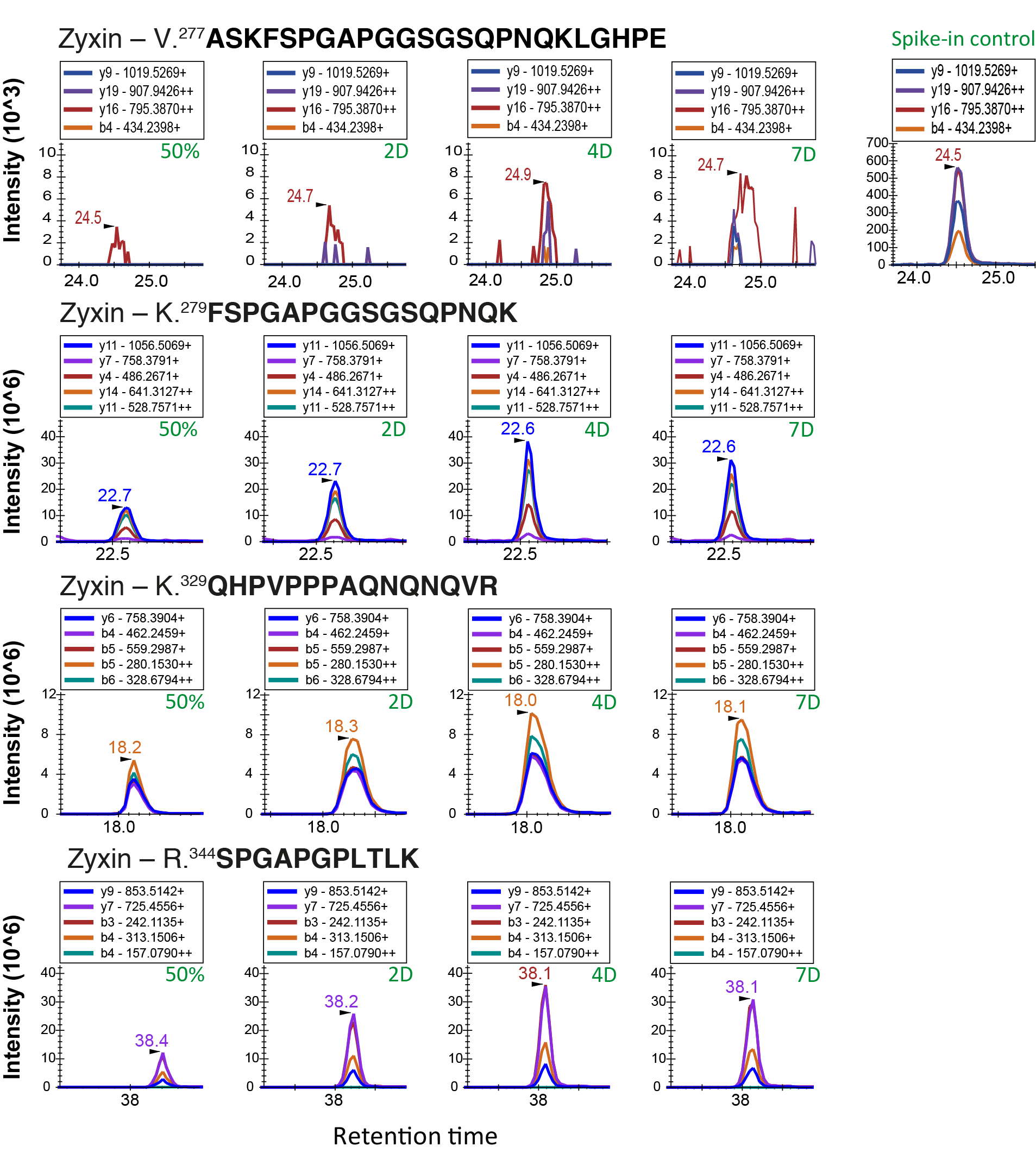

Supplement: Supplementary file 6 — Figure S5 [file 41419_2020_2883_MOESM6_ESM.png]

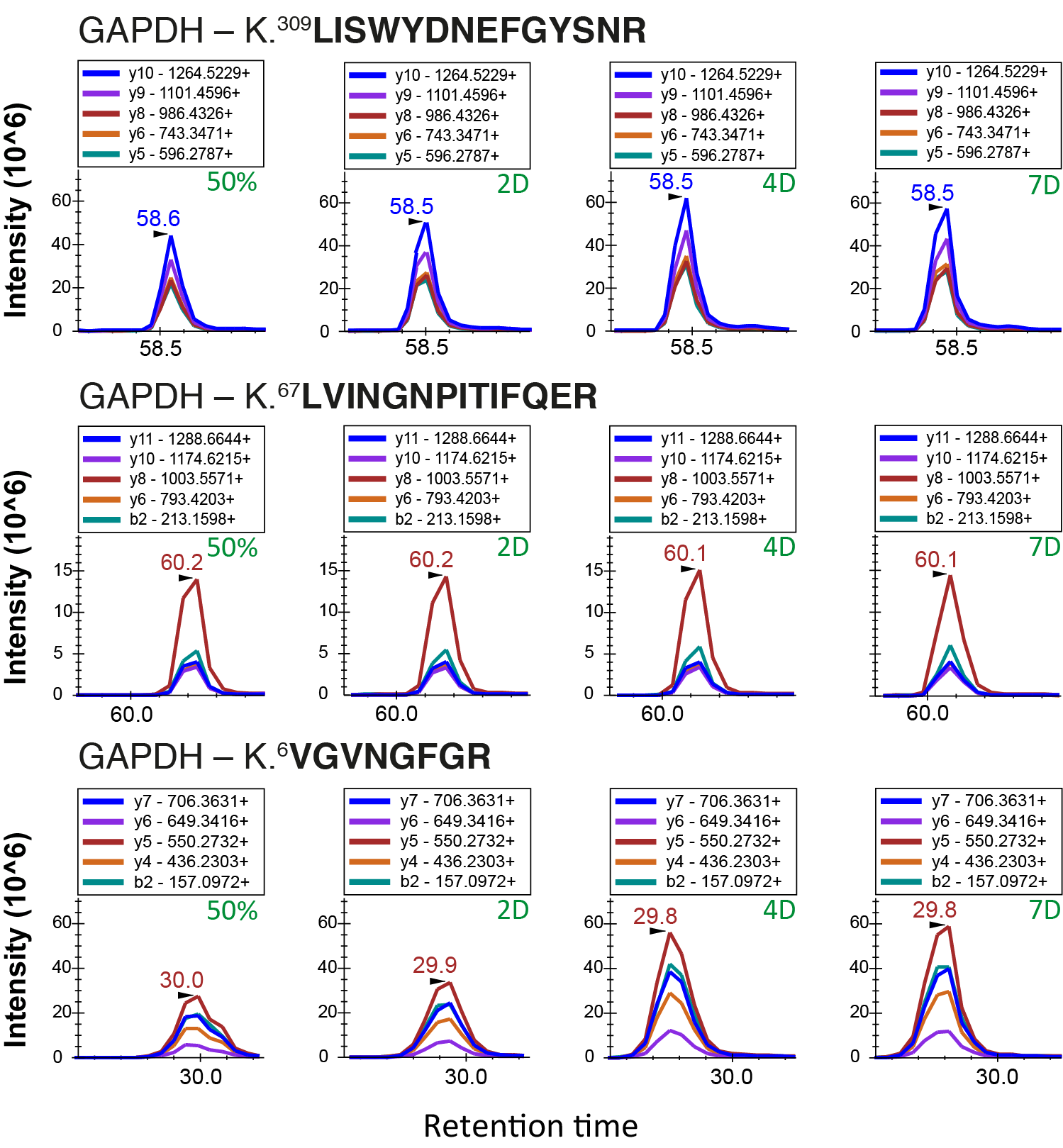

Supplement: Supplementary file 7 — Figure S6 [file 41419_2020_2883_MOESM7_ESM.png]

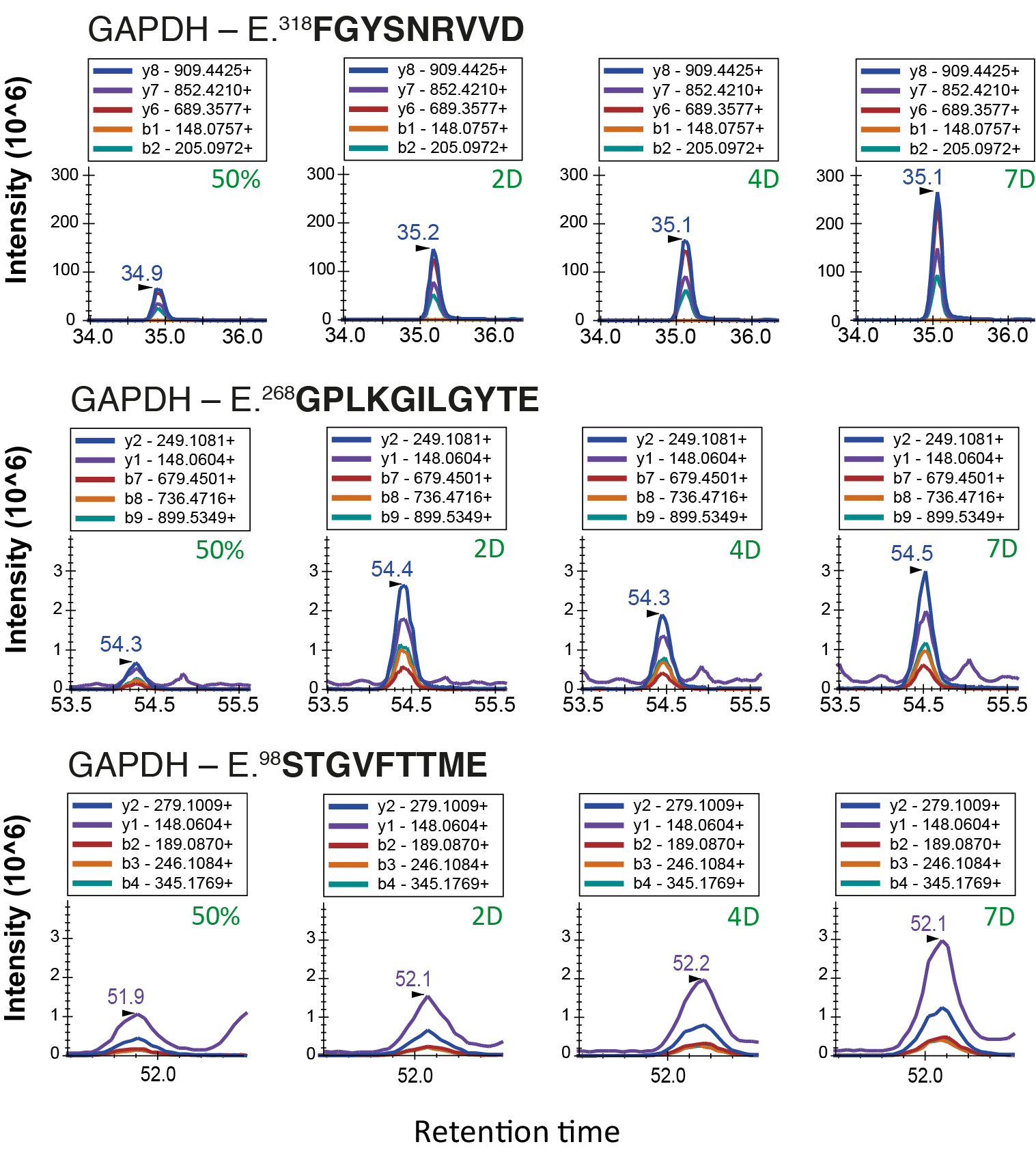

Supplement: Supplementary file 8 — Figure S7 [file 41419_2020_2883_MOESM8_ESM.png]

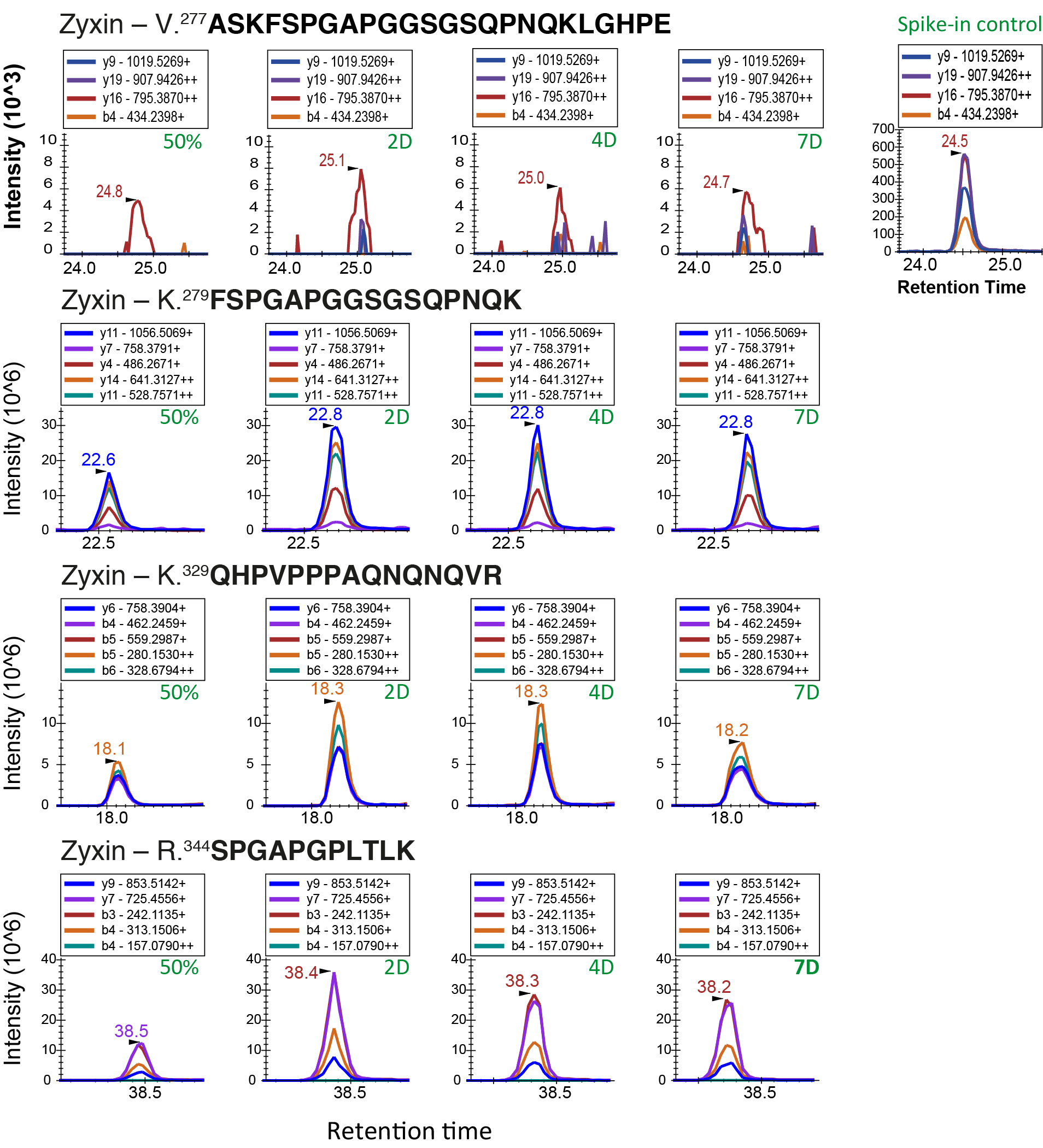

Supplement: Supplementary file 9 — Figure S8 [file 41419_2020_2883_MOESM9_ESM.png]

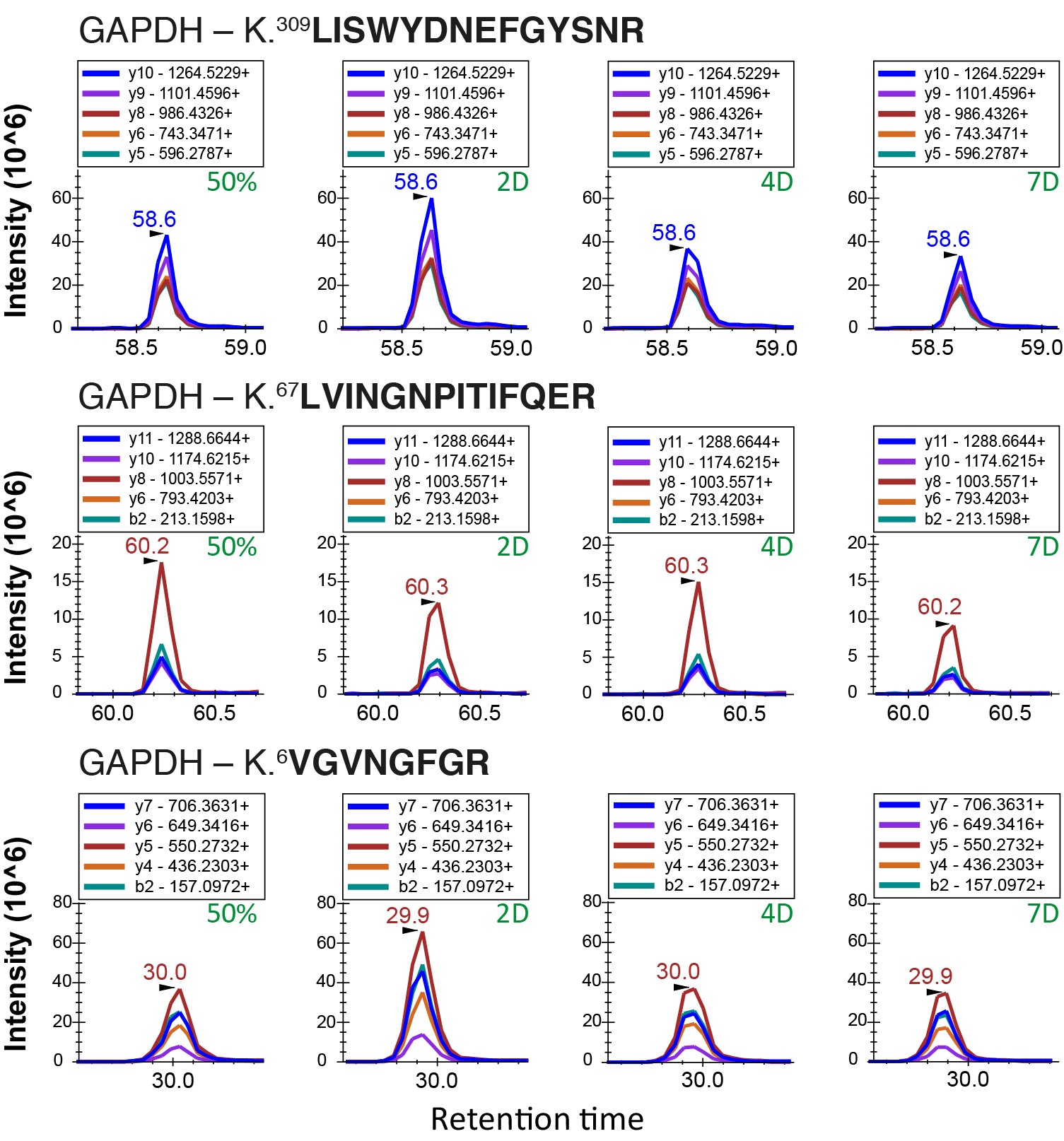

Supplement: Supplementary file 10 — Figure S9 [file 41419_2020_2883_MOESM10_ESM.png]

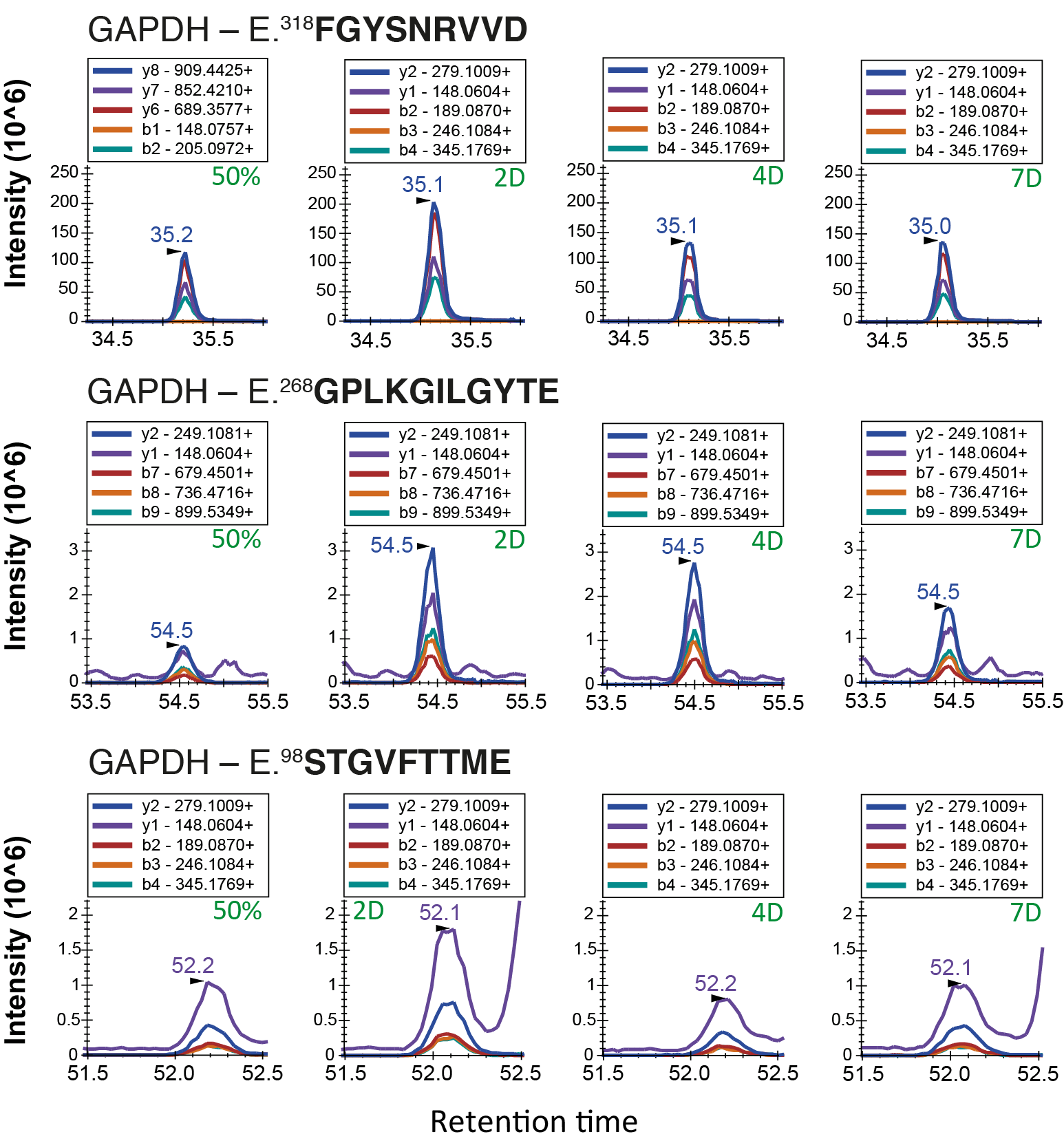

Supplement: Supplementary file 11 — Figure S10 [file 41419_2020_2883_MOESM11_ESM.png]
